# New Modified Deoxythymine with Dibranched Tetraethylene Glycol Stabilizes G-Quadruplex Structures

**DOI:** 10.3390/molecules25030705

**Published:** 2020-02-06

**Authors:** Hisae Tateishi-Karimata, Tatsuya Ohyama, Takahiro Muraoka, Shigenori Tanaka, Kazushi Kinbara, Naoki Sugimoto

**Affiliations:** 1 Frontier Institute for Biomolecular Engineering Research (FIBER), Konan University, 7-1-20 Minatojima-Minamimachi, Chuo-ku, Kobe 650-0047, Japan; tateishi@konan-u.ac.jp (H.T.-K.); t-ohyama@konan-u.ac.jp (T.O.); 2 Institute of Global Innovation Research, Tokyo University of Agriculture and Technology, 2-24-16 Naka-cho, Koganei, Tokyo 184-8588, Japan; muraoka@go.tuat.ac.jp; 3 Department of Computational Science, Graduate School of System Informatics, Kobe University, 1-1, Rokkodai, Nada-ku, Kobe 657-8501, Japan; tanaka2@kobe-u.ac.jp; 4 School of Life Science and Technology, Tokyo Institute of Technology, Nagatsuta-cho, Midori-ku, Yokohama 226-8501, Japan; kinbara.k.aa@m.titech.ac.jp; 5 Graduate School of Frontiers of Innovative Research in Science and Technology (FIRST), Konan University, 7-1-20 Minatojima-Minamimachi, Chuo-ku, Kobe 650-0047, Japan

**Keywords:** branched tetraethylene glycol, G-quadruplex, stability, modified nucleic acid, CH-π interactions

## Abstract

Methods for stabilizing G-quadruplex formation is a promising therapeutic approach for cancer treatment and other biomedical applications because stable G-quadruplexes efficiently inhibit biological reactions. Oligo and polyethylene glycols are promising biocompatible compounds, and we have shown that linear oligoethylene glycols can stabilize G-quadruplexes. Here, we developed a new modified deoxythymine with dibranched or tribranched tetraethylene glycol (TEG) and incorporated these TEG-modified deoxythymines into a loop region that forms an antiparallel G-quadruplex. We analyzed the stability of the modified G-quadruplexes, and the results showed that the tribranched TEG destabilized G-quadruplexes through entropic contributions, likely through steric hindrance. Interestingly, the dibranched TEG modification increased G-quadruplex stability relative to the unmodified DNA structures due to favorable enthalpic contributions. Molecular dynamics calculations suggested that dibranched TEG interacts with the G-quadruplex through hydrogen bonding and CH-π interactions. Moreover, these branched TEG-modified deoxythymine protected the DNA oligonucleotides from degradation by various nucleases in human serum. By taking advantage of the unique interactions between DNA and branched TEG, advanced DNA materials can be developed that affect the regulation of DNA structure.

## 1. Introduction

Methods for controlling biological reactions relevant to disease are very useful in therapeutic and medical applications [1]. The non-canonical structure of the G-quadruplex is often associated with cancer and neurodegenerative disease [2,3]. It has been reported that biological reactions such as transcription, translation, and telomerase elongation are inhibited by stable G-quadruplex formation in target genes [1,4,5,6]. To enhance the efficiency of G-quadruplex formation, small ligands that stabilize the G-quadruplex structure have been developed, such as 5,10,15,20-tetrakis-(*N*-methyl-4-pyridyl) porphyrin, berberine, and *N*-methyl mesoporphyrin IX [7,8,9]. These G-quadruplex-stabilizing compounds also stabilize duplex structures to some extent. Moreover, nucleic acids in cells are not sufficiently stable for practical therapeutic use, as these molecules are readily degraded by nucleases [10,11]. Therefore, simple and selective compounds that enable sequence-specific control of G-quadruplex formation and that resist nuclease degradation are desired.

**Figure 1:**
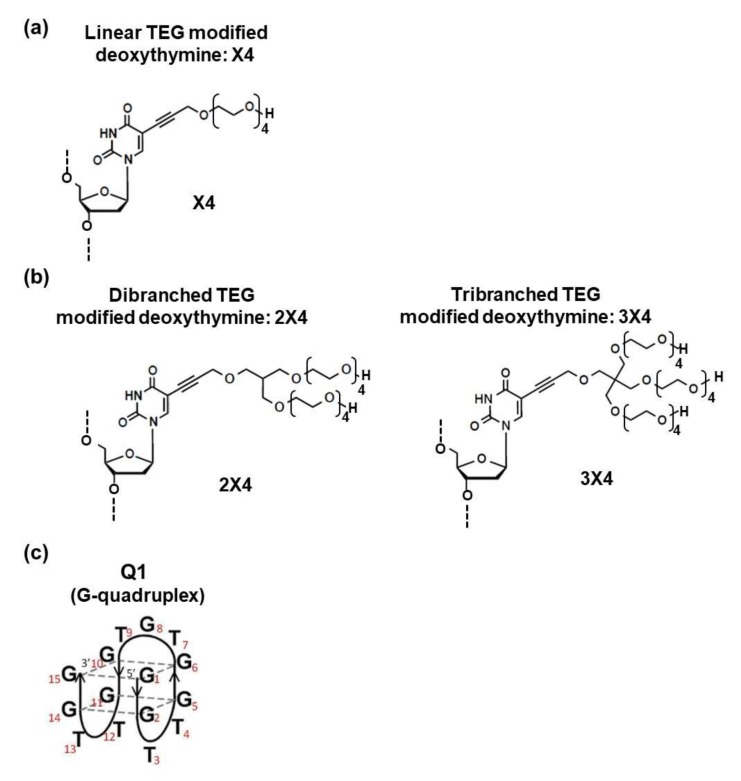
Chemical structures of (**a**) linear (X4), (**b**) dibranched (2X4), and tribranched (3X4) tetraethylene glycol (TEG)-modified deoxythymines. The linear TEG-modified deoxythymine (X4) was previously described [16,17]. (**c**) Schematic structure of the antiparallel G-quadruplex (Q1) used in this study.

Oligoethylene glycols and polyethylene glycols (PEGs) of various lengths are widely used in biotechnological applications. For example, PEG has been used to modify liposomes, nanoparticles, proteins, and nucleic acids to facilitate uptake into mammalian cells and enhance pharmacokinetics [12,13,14]. Moreover, PEGs provide nuclease resistance for nucleic acid structures through steric hindrance [15]. We recently demonstrated that an oligonucleotide with linear tetraethylene glycol (TEG)-modified deoxythymine (the main component of PEG200, average molecular weight of 200) (Figure 1a), formed intermolecular G-quadruplexes with single G-tract sequences [16]. Intermolecular G-quadruplexes induced by oligonucleotides containing TEG-modified deoxythymine were significantly more stable relative to unmodified DNA and efficiently inhibited reverse transcription [16]. Moreover, we found that TEG specifically stabilized G-quadruplexes via CH-π interactions between the nucleobases and the CH bonds of TEG [17]. Oligoethylene glycols covalently linked to DNA interact with G-quadruplexes and stabilize these DNA structures more significantly than free oligoethylene glycols in solution due to enthalpic contributions. Thus, to further enhance G-quadruplex stability, we developed novel modified deoxythymines with dibranched or tribranched TEG (Figure 1b) because branched TEGs have ethylene oxides that likely interact with the nucleobases of the G-quadruplexes. We report here the thermodynamic properties of the DNA structures adopted after modifications with branched deoxythymines (compared to unmodified DNA) and the effect of the TEG group on the nuclease resistance of DNA.

## 2. Results and Discussion

### 2.1. Sequence Design to Understand the Effect of Modified Deoxythymines on G-quadruplex Stability

We evaluated the effect of tetraethylene glycol on G-quadruplex stability. We synthesized deoxythymine tethered to dibranched (2X4) and tribranched (3X4) TEG as shown in Figure 1b,c, respectively. For accurate determination of thermodynamic parameters from thermal melting curves, DNA oligonucleotides must undergo a two-state transition between a single-stranded random coil and a structured form [18]. Although G-rich sequences show a significant degree of structural polymorphism, the thrombin aptamer sequence reliably folds into an antiparallel G-quadruplex that melts in a two-state manner. Thus, we used the thrombin aptamer (Q1) as a model G-quadruplex (Figure 1c) and incorporated the modified deoxythymines into this aptamer (Table 1). Based on a previous NMR study of the thrombin aptamer, deoxythymines in position four (T_4_) and 13 (T_13_) stack on a G-quartet, while deoxythymine in position seven (T_7_) does not [19,20]. We proved that stabilization of G-quadruplexes by linear TEG-modified deoxythymines depends on the positions of incorporation; deoxythymines stacked on a G-quartet significantly increase stability, while those not stacked show almost no effect [17]. Thus, we replaced deoxythymines in the loop regions of Q1 with (2X4) or (3X4) as shown in Table 1. In our nomenclature, the type of modification is followed by the position number; for example, Q1-(2X4)_7_ indicates the thrombin-binding aptamer Q1 with dibranched TEG-modified deoxythymine (2X4) at position seven.

### 2.2. Thermodynamic Analysis of the Effects of TEG-modified Deoxythymines on G-quadruplex Stability

**Figure 2:**
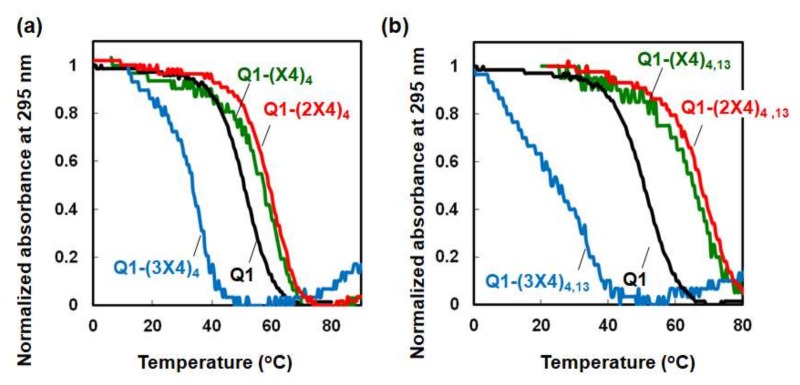
Normalized UV melting curves for (**a**) Q1 (black), Q1-(X4)_4_ (green), Q1-(2X4)_4_ (red), and Q1-(3X4)_4_ (blue) and (**b**) Q1 (black), Q1-(X4)_4__,13_ (green), Q1-(2X4)_4__,13_ (red), and Q1-(3X4)_4__,13_ (blue). The buffer contained 100 mM KCl, 10 mM K_2_HPO_4_ (pH 7.5 at 25 °C), and 1 mM K_2_EDTA. The strand concentration was 3 μM.

To analyze how the stability of the DNA structures was affected by the TEG modification, the melting temperature (*T*_m_) was determined (Figure 2). The *T*_m_ values for 3 μM Q1, Q1-(X4)_4_, Q1-(2X4)_4_, and Q1-(3X4)_4_ were 50.7, 58.8, 61.6, and 36.4 °C, respectively. Interestingly, the *T*_m_ values for linear and dibranched TEG increased relative to the unmodified G-quadruplex; however, tribranched TEG decreased significantly. Moreover, G-quadruplexes with two modified deoxythymines gave *T*_m_ values of 3 μM Q1-(X4)_4,13_ and Q1-(2X4)_4,13_ of 66.4 and 70.1 °C, respectively. The *T*_m_ value of Q1-(3X4)_4,13_ could not be calculated because the melting curve of Q1-(3X4)_4,13_ did not show a clear thermal transition. Thus, the incorporation of certain TEG-modified deoxythymines caused significant stabilization with increasing number of TEGs the G-quadruplex.

To understand how the branched TEG modifications altered the stability of the DNA structures, we evaluated the effect of the position of the dibranched TEG-modified deoxythymines on the thermodynamic parameters of G-quadruplex formation (Table 2).

The deoxythymines at positions four and 13 in the thrombin aptamer stack on the G-quartet, but deoxythymine in position seven does not [21]. We determined the free energy changes (∆*G*°_25_) upon structure formation using oligonucleotides with branched TEG. The ∆*G*°_25_ values of Q1-(2X4)_4_ and Q1-(2X4)_7_ were –5.4 and –3.7 kcal·mol^−1^, respectively. Significantly more stabilization was observed when the dibranched TEG-modified deoxythymines were in a position in which stacking was observed in the NMR study. Thus, the stacking interactions between deoxythymine and dibranched TEG is important for stabilization of the G-quadruplex. The greater stabilization of Q1-(2X4)_4_ relative to Q1-(2X4)_7_ was due to a favorable enthalpic contribution (Table 2). Importantly, Q1-(2X4)_4,13_, which had two substitutions, was more stable than the singly substituted G-quadruplexes and showed a more favorable enthalpic contribution. These enthalpic contributions have been previously observed [22,23,24] and are due to the formation of stacking interactions between the terminal base pair and the dangling ends via π-π interactions [22,25]. Moreover, it has also been reported that the carbohydrates stack on the ends of the DNA duplexes via CH-π interactions, stabilizing the duplexes [26]. We have also shown that the presence of linear TEG in the loop of the G-quadruplex allows adoption of a conformation suitable for CH-π interactions of TEG with a G-quartet [17]. Thus, the stabilization of G-quadruplexes by dibranched TEGs may also be due to CH-π interactions. Contrastingly, the ∆*G*°_25_ value of Q1-(3X4)_4_ was greater than that of Q1-(2X4)_4_, with a significantly lower *T*∆*S*° value. Because tribranched TEG is likely too large to fit inside the two lateral loops (T_3_–T_4_ and T_12_–T_13_) of Q1 (Figure 1b), the stacking interactions between the bases in the lateral loops of the G-quartets would collapse. Therefore, dibranched TEGs are optimal for stabilizing G-quadruplexes.

### 2.3. Molecular Dynamic (MD) Calculations for G-quadruplexes with Modified Deoxythymines

To understand the detailed mechanism by which dibranched TEGs stabilize the G-quadruplex, we carried out 200-ns molecular dynamic (MD) calculations using Q1-(2X4)_4_ (see Supporting Information). For reference, MD calculations were also performed on the structure of the unmodified G-quadruplex of Q1 and the linear TEG-modified G-quadruplex of Q1-(X4)_4_. Our previous MD calculations using a linear TEG-modified G-quadruplex did not consider the influence of KCl [17]. Thus, the calculation performed in this study accounted for 100 mM KCl because K^+^ and Cl^–^ ions likely affect the G-quadruplex structure.

**Figure 3:**
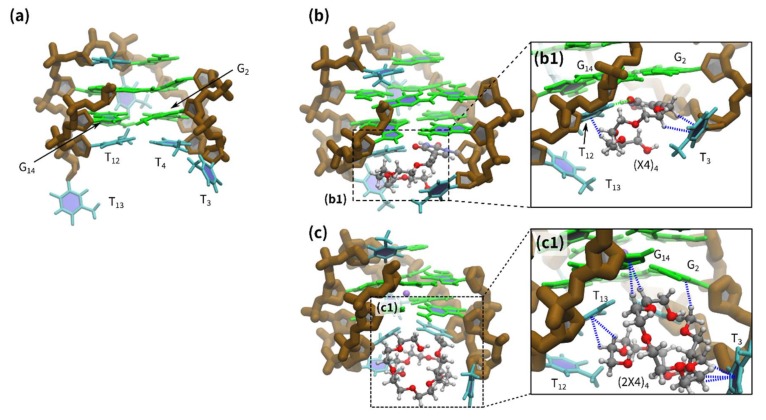
Representative structures of (**a**) Q1 at 50 ns, (**b**) Q1-(X4)_4_ at 34 ns, and (**c**) Q1-(2X4)_4_ at 133 ns and (b1 and c1) enlarged views of the dashed squares. Green, cyan, and brown illustrate guanine, thymine, and the backbone of the G-quadruplex, respectively. The red, blue, gray, and white colors of the ball-and-stick models illustrate oxygen, nitrogen, carbon, and hydrogen atoms, respectively. The expected hydrogen bond and CH-π interactions between TEG and the oligonucleotide are indicated by green and blue dashed lines, respectively.

We sampled 18,000 (20 to 200 ns) snapshots from MD trajectories in an attempt to understand the TEG-induced stabilization. These snapshots were selected where the root mean square deviation (RMSD) values for the G-quadruplex backbone reached equilibrium state (Figure S1). The thymine bases, including T_4_ in the Q1 lateral loops, were stacked in the G-quartet (Figure 3a). Several types of TEG conformations were observed in the 20–200 ns period. Thus, snapshots taken during the MD simulations were classified into 10 groups (clusters) depending on the RMSD values for all atoms of linear and dibranched TEGs, using the hierarchical agglomerative clustering method (Figures S2 and S3). Figure 3b,c show the major structures of linear and dibranched TEG, respectively. The linear TEG in Q1-(X4)_4_ collapsed and was located near the G-quartet, between the lateral loops (Figure 3b). The structural analysis showed that the linear TEG interacts with the base of the loop facing the T_4_ base and stabilizes the facing loop to bridge the two lateral loops (Figure 3b). The linear TEG structure is consistent with our previous study [17]. In Figure 3c, structural analysis shows dibranched TEG in Q1-(2X4)_4_ interacting with G_2_, T_3_, T_13_, and G_14_ via CH-π interactions (Figure 3c1). The CH of TEG in Q1-(2X4)_4_ is located close to G_2_, T_3_, T_13_, and G_14_, suggesting CH-π interactions (Figure 3c1, blue dashed line). Linear TEG can interact only in one direction; in contrast, dibranched TEG can access bases in multiple directions, greatly increasing stabilization efficiency.

**Figure 4:**
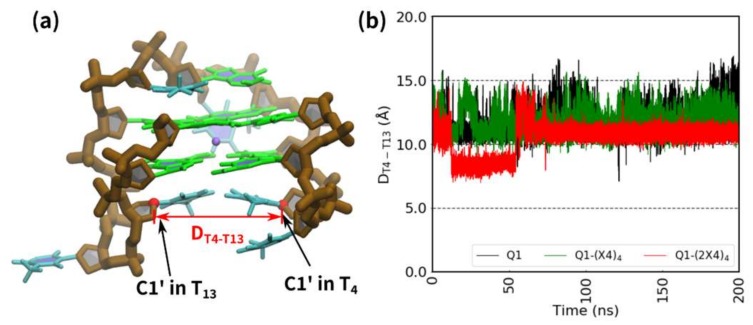
Fluctuation of loop regions in the G-quadruplexes. (**a**) Positions of the C1′ atoms of Q1 are annotated, and the distance between these atoms is indicated by a red arrow. Brown, green, and cyan sticks show the backbone, guanine bases, and thymine bases, respectively. The purple sphere represents a potassium cation. (**b**) Change in distance between the C1′ atoms (T_4_–T_13_) in Q1 (black), Q1-(X4)_4_ (green), and Q1-(2X4)_4_ (red).

To confirm the effect of TEG on the loop bases, we measured the distance between the C1′ atoms of the T_4_ and T_13_ nucleotides [D_T4-T13_ (Å), Figure 4a]. Figure 4b shows the time course of D_T4–T13_ for Q1, Q1-(X4)_4_, and Q1-(2X4)_4_. During the 200 ns MD simulation, D_T4-T13_ for Q1 and Q1-(X4)_4_ fluctuated in the range of 10–15 Å. However, Q1-(2X4)_4_ showed an almost constant value around 8 Å between 15 ns and 50 ns, with only minor fluctuation. After 50 ns, the distance of Q1-(2X4)_4_ maintained with small fluctuations around 10.8 Å. This result suggests that the loop structure of Q1-(2X4)_4_ is stabilized. Thus, TEG is thought to stabilize the G-quadruplex by stabilizing the lateral loop of the G-quadruplex.

**Figure 5:**
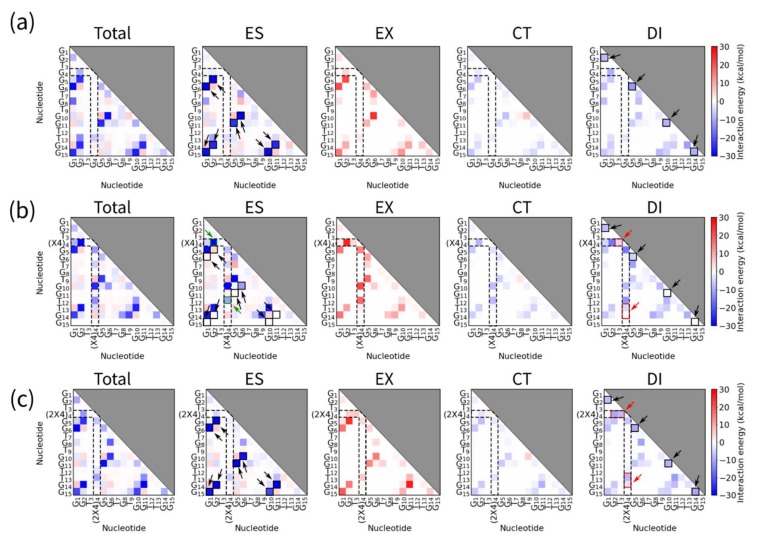
Total electrostatic (ES), exchange repulsion (EX), charge transfer (CT), and dispersion force (DI) energy graphs are shown, indicating the interaction energies between each base in (**a**) Q1, (**b**) Q1-(X4)_4_, and (**c**) Q1-(2X4)_4_. Interactions of T_4_, (X4)_4_, or (2X4)_4_ with other bases were highlighted by broken lines. Blue squares with black arrows in ES and DI indicate hydrogen bonds and stacking interactions in G-quartets, respectively. Blue squares with green arrows in ES and with red arrows in DI show hydrogen bonds and CH-π interactions of (X4) and (2X4) with bases of G-quadruplexes, respectively.

We also estimated the total electrostatic (ES), exchange repulsion (EX), charge transfer (CT), and dispersion force (DI) energies to confirm quantitatively the interactions found in the structural analysis. Graphs showing interaction energies are shown in Figure 5. Interactions between T_4_ and other bases are highlighted by dashed lines. Blue squares with black arrows in ES and DI indicate hydrogen bonds and stacking interactions in G-quartets, respectively. In the ES graphs for Q1-(X4)_4_, there are dark blue squares in the dashed line (Figure 5b, ES graphs, green arrows). These blue squares show the hydrogen bond between T_12_ and thymine base in (X4)_4_ in the vicinity. In contrast, the dark blue squares in the DI graphs represent CH-π interactions (Figure 5b,c, DI graphs, red arrows). The results indicated that linear and dibranched TEGs stabilized G-quadruplex hydrogen bond and CH-π interactions.

### 2.4. Modified Bases Improve Nuclease Resistance

Dibranched TEG acts as a cover for the single-stranded region (loop). Because this single-stranded region is a potential nuclease cleavage initiation site, the TEG modification is thought to enhance the nuclease resistance of the DNA.

**Figure 6:**
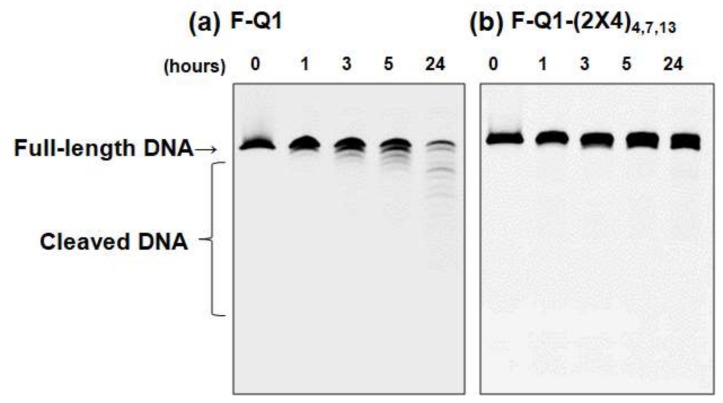
Denaturing gel electrophoresis of (**a**) F-Q1 and (**b**) F-Q1-(2X4)_4,,__7,1__3_ after the addition of human serum at 37 °C. Samples incubated for 0, 1, 3, 5, and 24 h were loaded on lanes one to five, respectively.

To determine whether dibranched TEG-modified deoxythymine shows increased nuclease resistance, we incorporated dibranched TEG-modified deoxythymines into G-quadruplexes. To quantify nuclease resistance, the oligonucleotides were fluorescently modified at the 5′ end and named F-Q1 and F-Q1-(2X4)_4,7,13_, corresponding to oligonucleotides without and with dibranched TEG, respectively (Table 1). Solutions (1 μM final concentration) of F-Q1 and F-Q1-(2X4)_4,7,13_ were prepared in 100 mM KCl, and nine volumes of serum containing nucleases was added. The reaction was stopped by adding ten volumes of formamide solution and heating to 95 °C for 15 min to deactivate the nucleases. Degradation of the oligonucleotides was examined after separation using denaturing PAGE. The change of oligonucleotides length due to degradation was also confirmed by size marker DNA (Figure S4). As shown in Figure 6a, F-Q1 degraded over time at 37 °C. After 24 h of incubation, full-length F-Q1 degraded more than 80% (Figure 5a, lane 5). In contrast, degradation of F-Q1-(2X4)_4,9,13_ was markedly inhibited (Figure 6b, lanes one to five). It has been reported that nucleobases with long amphiphilic functional groups are resistant to nuclease cleavage due to steric hindrance when compared to natural DNA [27]. Dibranched TEG-modified deoxythymine protected the DNA from nuclease degradation and contributed to significant stabilization of the G-quadruplex structure. As shown in the MD calculations, dibranched TEG surrounded the G-quadruplex due to interactions between the DNA bases and dibranched TEG; these interactions should prevent nuclease access. We considered that tribranched TEG may show the large steric hindrance and high nuclease resistance. Thus, we estimated the nuclease resistance for F-Q1-(3X4)_4,7,13_. Interestingly, the oligonucleotide with tribranched TEGs showed higher nuclease resistance than the oligonucleotide with dibranched TEGs (Figure S5), indicating that steric hindrance of branched TEGs suppressed nuclease binding. Thus, these branched TEG-modified deoxythymine would be useful in applications because these modifications also protect the oligonucleotide from nucleases.

## 3. Materials and Methods

### 3.1. Chemicals and Materials

All oligodeoxynucleotides used in this study, except for oligodeoxynucleotides modified with oligoethylene glycols, were purified by high performance liquid chromatography by the vendor, Japan Bio Services Co. LTD. (Saitama, Japan), Japan Single-strand concentrations of DNA oligonucleotides were calculated from the absorbance measured at 260 nm and 90 °C using single-strand extinction coefficients calculated from the mononucleotide and dinucleotide data according to the nearest-neighbor approximation model [28]. Absorbance was measured using a Shimadzu 1700 spectrophotometer connected to Shimadzu UV-1700 or UV-1800 spectrophotometers equipped with a temperature controller (Shimadzu, Kyoto, Japan). The amidite of TEG-modified deoxythymine was synthesized following a reported procedure (see Supporting Information) [16].

### 3.2. Thermodynamic Analysis

Ultraviolet (UV) absorbance was measured on Shimadzu UV-1700 or UV-1800 spectrophotometers equipped with a temperature controller (Shimadzu, Kyoto, Japan). Melting curves were measured at 295 nm in buffer containing 100 mM KCl, 10 mM K_2_HPO_4_ (pH 7.0 at 37 °C), and 1 mM K_2_EDTA. Samples were heated at a rate of 0.5 °C min^−1^. Using the analysis method of the unimolecular transition described previously [29], we analyzed the melting curves in this study. Thermodynamic parameters (Table 2) were calculated using average values obtained from curve fitting at different DNA concentrations (2, 3, and 5 μM) [29]. Prior to measurement, DNA samples were heated to 95 °C, cooled to 0 °C at a rate of −0.5 °C min^−1^, and incubated at 0 °C for 30 min.

### 3.3. Molecular Dynamic (MD) Simulations

The NMR structure of anti-parallel type G-quadruplex with 5′-GGTTGGTGTGGTTGG-3′ was obtained from Protein Data Bank (PDB ID 1C35) [30] as the initial structure of Q1 in our simulations. Since the structure has trajectory of potassium cations between G-quartets, we adopted the first coordinate in trajectory and remove other coordinates. Based on structure of Q1, Q1-(2X4)_4_, and Q1-(3X4)_4_ were prepared by modifying methyl group of fourth deoxythymine base in Q1. Dibranched TEG modified deoxythymine moieties was built and merged with Q1 by Discovery Studio Ver. 2017 [31]. To neutralize the system charge, fourteen cations were placed around phosphate groups of Q1. We set the simulation box size to 6.800 Å by 6.200 Å by 6.763 Å to make distance between box edges and G-quadruplex at least 20 Å. The boxes were filled up by TIP3P water molecules [32], and added seventeen potassium and chloride ions, respectively, corresponding to 100 mM KCl solution. Molecular dynamics simulations for these systems were performed by GROMACS Ver. 5.1.2 [33]. The force field AMBER14SB was applied to nucleotides [34]. The force fields for dibranched TEG modified deoxythymine was generated based on General AMBER force field (GAFF) by antechamber and residuegen modules in AmberTools [35,36]. The partial atomic charges of these TEG modified deoxythymine were determined by restrained electrostatic potential (RESP) calculation [37] using Gaussian09 software [38]. These force fields for AMBER formats were converted by ACPYPE program [39,40]. For these systems, solution atoms were optimized by 10,000 steps with harmonic position restraints of heavy atoms of DNAs with a force constant of 4184 kJ mol^−1^ nm^−2^, and whole atoms were optimized by 10,000 steps. Solution atoms were gradually heated to 310 K for 100 ps with harmonically restrained under the NVT ensemble. Equilibration simulations were run for 1 ns at 310 K under NVT and NPT ensemble, respectively. Finally, NPT ensemble simulations were carried out for 200 ns at 310 K and 1 atm. In all simulations, timestep was set to 2 fs and all covalent bonds with hydrogen atoms were constrained using LINCS algorithm [41]. We used periodic boundary condition and particle mesh Ewald (PME) method to treat long-range electrostatic interactions with a cutoff of 1.0 nm [42,43].

### 3.4. Assays for Nuclease Resistance

The chemical stability of oligonucleotides was analyzed in the presence of 90% (*v/v*) human serum. We have purchased a human serum type AB (Lonza), which contains probably various nucleases such as endonucleases and exonucleases. The degradation of 1 μM DNAs by nucleases present in the serum was examined after various incubation times. Reactions were quenched at the indicated times by the addition of a 10-fold excess volume of stop solution (80 wt% formamide, 10 mM Na2EDTA, and 0.1% blue dextran) and incubated at 95 °C for 15 min. The oligonucleotides were separated by denaturing PAGE and fluoresce levels of the DNA with fluorescent dye were quantified with a fluorescent imager (GE Helthcare, FLA-5100). Before the denaturing PAGE, the precipitate of proteins in the serum was removed by centrifugation. The gel electrophoresis was carried out on 10% nondenaturing polyacrylamide gels at 25 °C.

## 4. Conclusions

In this study, we developed a new modified deoxythymine with dibranched or tribranched TEG and incorporated these modified bases into an antiparallel G-quadruplex. We analyzed the stability of the resulting G-quadruplexes and found that tribranched TEG destabilized G-quadruplexes through entropic contributions, likely due to steric hindrance by the tribranched tetraethylene glycol. Interestingly, the dibranched TEG modification increased G-quadruplex stability relative to unmodified DNA structures due to favorable enthalpic contributions. MD calculations suggested that dibranched TEG interacts with phosphates in the G-quadruplex backbone structure through hydrogen bonding and CH-π interactions. Moreover, dibranched TEG-modified deoxythymine protected the DNA oligonucleotides from degradation by various nucleases in human serum. Our results are applicable for designing and controlling DNA structures within the cell to regulate biological reactions via non-canonical structure formation.

## Figures and Tables

**Table 1:** DNA sequences used in this study.

Name	Sequences (5′-3′) ^a^
Q1	GGTTGGTGTGGTTGG
Q1-(X4)_4_	GGT(X4)GGTGTGGTTGG
Q1-(X4)_4,13_	GGT(X4)GGTGTGGT(X4)GG
Q1-(2X4)_4_	GGT(2X4)GGTGTGGTTGG
Q1-(2X4)_7_	GGTTGG(2X4)GTGGTTGG
Q1-(2X4)_4,13_	GGT(2X4)GGTGTGGT(2X4)GG
Q1-(3X4)_4_	GGT(3X4)GGTGTGGTTGG
Q1-(3X4)_4,13_	GGT(3X4)GGTGTGGT(3X4)GG
F-Q1 ^b^	F-GGTTGGTGTGGTTGG
F-Q1-(2X4)_4,__7,__13_^b^	F-GGT(2X4)GG(2X4)GTGGT(2X4)GG
F-Q1-(3X4)_4,__7,__13_^b^	F-GGT(3X4)GG(3X4)GTGGT(3X4)GG

^a^ Loop regions in the G-quadruplex are underlined; ^b^ F indicates 6-carboxyfluorescein.

**Table 2:** Thermodynamic parameters of G-quadruplex formation with branched TEGs ^a^.

Sequence	∆ *H*° (kcal mol^−1^)	*T*∆*S*° (kcal mol^−1^)	∆*G*°_25_ (kcal mol^−1^)	*T*_m_^b^ (°C)
Q1-(2X4)_4_	−45.3 ± 3.3	−39.9 ± 2.9	−5.4 ± 0.4	61.6
Q1-(2X4)_7_	−38.2 ± 8.7	−34.5 ± 1.6	−3.7 ± 0.7	53.2
Q1-(2X4)_4,13_	−50.5 ± 1.5	−43.4 ± 5.4	−7.1 ± 0.1	70.1
Q1-(3X4)_4_	−72.9 ± 3.3	−69.7 ± 2.9	−3.2 ± 0.4	36.4
Q1-(3X4)_4,13_		n.d.		

^a^ All experiments were carried out in buffer containing 100 mM KCl, 10 mM K_2_HPO_4_ (pH 7.0), and 1 mM K_2_EDTA. Thermodynamic parameters were calculated (n.d., not determined) from the average values obtained from curve fitting; ^b^ The melting temperature was calculated using a strand concentration of 3 µM.

## Supplementary Materials

The following are available online at https://www.mdpi.com/1420-3049/25/3/705/s1.
