# Supplementary material for: New Modified Deoxythymine with Dibranched Tetraethylene Glycol Stabilizes G-Quadruplex Structures
Source: Molecules. 2020 Feb 6;25(3):705. doi: 10.3390/molecules25030705 (PMC7036917; doi:10.3390/molecules25030705)
Supplement: Supplementary file 1 [file molecules-25-00705-s001.pdf]

Supporting Information

New modified deoxythymine with dibranched tetraethylene glycol stabilizes G-quadruplex structures

Hisae Tateishi-Karimata[1], Tatsuya Ohyama[1], Takahiro Muraoka[2], Shigenori Tanaka[3], Kazushi Kinbara[4], and Naoki Sugimoto[1, 5] *

[1] Frontier Institute for Biomolecular Engineering Research (FIBER), Konan University, 7-1-20 Minatojima-Minamimachi, Chuo-ku, Kobe 650-0047, Japan

[2] Institute of Global Innovation Research, Tokyo University of Agriculture and Technology, 2-24-16 Naka-cho, Koganei, Tokyo 184-8588, Japan

[3] Department of Computational Science, Graduate School of System Informatics, Kobe University, 1-1, Rokkodai, Nada-ku, Kobe, 657-8501, Japan

[4] School of Life Science and Technology, Tokyo Institute of Technology, Nagatsuta-cho, Midori-ku, Yokohama 226-8501, Japan

[5] Graduate School of Frontiers of Innovative Research in Science and Technology (FIRST), Konan University, 7-1-20 Minatojima-Minamimachi, Chuo-ku, Kobe, 650-0047, Japan

* Correspondence: sugimoto@konan-u.ac.jp;

MATERIALS AND METHODS

Synthesis of Dibranched TEG modified deoxythymine

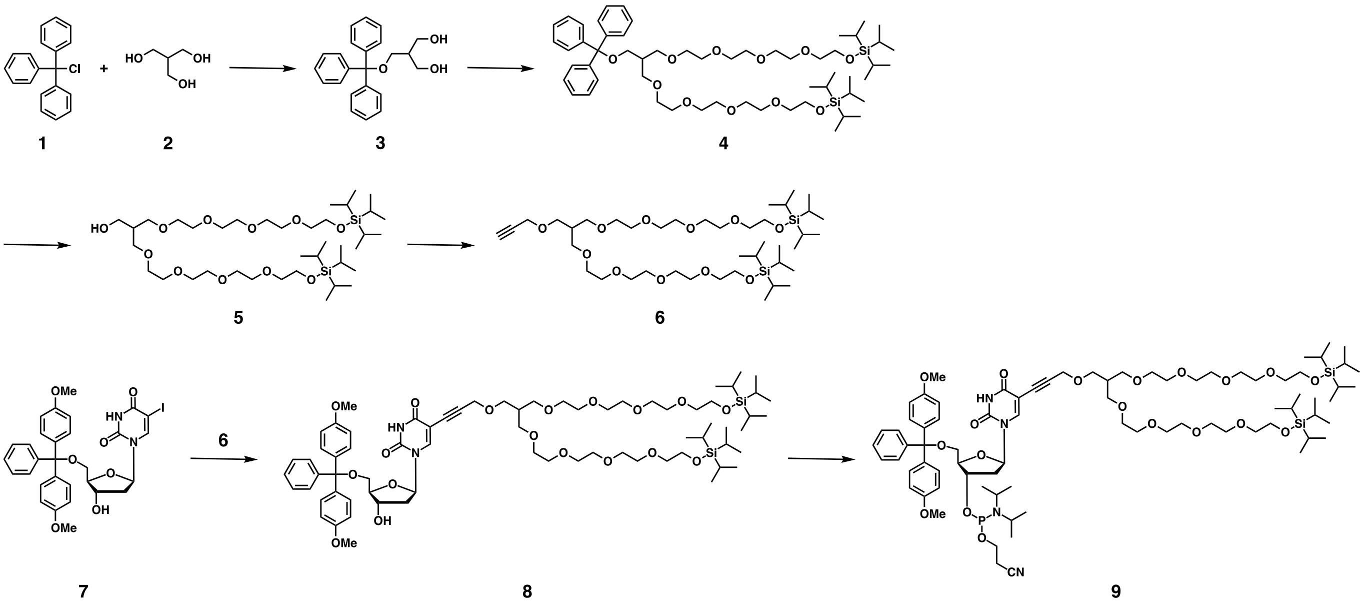

Synthesis of 3. To a dry pyridine (10 mL) solution of **2** (0.952 g, 8.97 mmol) was added **1** (1.23 g, 4.41 mmol) at 25 °C under A, and the resulting mixture was stirred overnight at 25 °C. To the reaction mixture was added water (100 mL), and the resulting mixture was extracted with EtOAc (100 mL, three times). The collected organic extract was washed with brine (50 mL) and dried over anhydrous Na_2SO_4, and filtered off from insoluble substances. The filtrate was evaporated to dryness under reduced pressure at 30 °C, and the residue was chromatographed on silica gel (Silica Gel 60) with a gradient of AcOEt/*n*-hexane (20/80 to 60/40 v/v) to allow isolation of **3** (1.79 g, 3.38 mmol) as white solid in 77% yield. ^{1}H NMR (400 MHz, $CDCl_3$ containing 0.03% TMS, 23 °C): δ 7.42 (m, 6H), 7.30 (m, 6H), 7.24 (m, 3H), 3.81 (t, J = 5.4 Hz, 4H), 3.28 (d, J = 5.4 Hz, 2H), 2.03 (m, 3H) ppm. ESI-TOF MS: *m*/*z*: calculated for $C_{23}H_{24}NaO_3$: 371.1623 [M + Na]$^+$; found: 371.1635. Melting point: 94.9–97.8 °C.

Synthesis of 4. To a dry THF (10 mL) suspension of NaH (washed twice with dry *n*-hexane to remove mineral oil just prior to use; 411 mg, 17.1 mmol) was added a dry THF (45 mL) solution of 3,3-diisopropyl-2-methyl-4,7,10,13-tetraoxa-3-silapentadecan-15-yl 4-methylbenzenesulfonate (TIPS-TEG-OTs, 3.50 g, 6.94 mmol) dropwise over 30 min at 25 °C under Ar, and the resulting mixture was refluxed. To the resulting mixture was added **3** (1.13 g, 3.23 mmol), and the reaction mixture was refluxed overnight. Then, the

reaction mixture was cooled to 0 °C followed by addition of water (5 mL) and CH_2Cl_2 (200 mL), and the resulting mixture was filtrated through celite 545. The filtrate was evaporated to dryness under reduced pressure at 40 °C, and the residue was chromatographed on silica gel (Silica Gel 60) with a gradient of AcOEt/*n*-hexane (20/80 to 30/70 v/v) to allow isolation of **4** (1.68 g, 1.66 mmol) as colorless oil in 51% yield. ^{1}H NMR (400 MHz, $CDCl_3$ containing 0.03% TMS, 23 °C): δ 7.42 (m, 6H), 7.29 (m, 6H), 7.23 (m, 3H), 3.83 (t, J = 5.6 Hz, 4H), 3.7–3.5 (m, 32H), 3.15 (d, J = 5.6 Hz, 2H), 2.19 (m, 1H), 1.10–1.05 (m, 42H) ppm. ESI-TOF MS: *m*/*z*: calculated for $C_{57}H_{96}NaO_{11}Si_2$: 1035.6389 $[M + Na]^+$; found: 1035.6402.

Synthesis of 5. To a dry MeOH (60 mL) solution of **4** (1.48 g, 1.46 mmol) was added glacial acetic acid (10 mL) dropwise over 20 min at 25 °C under Ar, and the resulting mixture was refluxed for 3 h. Then, the reaction mixture was cooled to 25 °C followed by evaporation under reduced pressure at 40 °C. To the residue was added CH_2Cl_2 (200 mL), and the mixture was washed with brine (200 mL, three times). The collected organic extract was dried over anhydrous Na_2SO_4, and filtered off from insoluble substances. The filtrate was evaporated to dryness under reduced pressure at 40 °C, and the residue was chromatographed on silica gel (Silica Gel 60) with a gradient of AcOEt/*n*-hexane (20/80 to 100/0 v/v) to allow isolation of **5** (0.740 g, 0.959 mmol) as colorless oil in 66% yield. ^{1}H NMR (400 MHz, $CDCl_3$ containing 0.03% TMS, 29 °C): δ 3.83 (t, J = 5.8 Hz, 4H), 3.74 (dd, J = 4.8 and 5.8 Hz, 2H), 3.7–3.5 (m, 32H), 2.87 (t, J = 4.8 Hz, 1H), 2.13 (sept, J = 5.8 Hz, 1H), 1.08–1.03 (m, 42H) ppm. ESI-TOF MS: *m*/*z*: calculated for $C_{38}H_{82}NaO_{11}Si_2$: 793.5293 $[M + Na]^+$; found: 793.5271.

Synthesis of 6. To a dry THF (10 mL) suspension of NaH (washed twice with dry *n*-hexane to remove mineral oil just prior to use; 57 mg, 2.5 mmol) was added a dry THF (20 mL) solution of **5** (0.733 g, 0.950 mmol) dropwise over 30 min at 0 °C under Ar, and the resulting mixture was stirred for 10 min at 0 °C. To the reaction mixture was added propargyl bromide (0.100 mL, 1.33 mmol) at 0 °C, and the resulting mixture was stirred overnight at 25 °C. Then, the reaction mixture was cooled to 0 °C followed by addition of water (1 mL) and CH_2Cl_2 (50 mL), and the resulting mixture was filtrated through celite 545. The filtrate was evaporated to dryness under reduced pressure at 40 °C, and the residue was chromatographed on silica gel (Silica Gel 60) with a gradient of AcOEt/*n*-hexane (50/50 to 70/30 v/v) to allow isolation of **6** (0.602 g,

0.745 mmol) as colorless oil in 78% yield. ^{1}H NMR (400 MHz, $CDCl_3$ containing 0.03% TMS, 24 °C): δ 4.11 (d, J = 2.4 Hz, 2H), 3.83 (t, J = 5.6 Hz, 4H), 3.7–3.5 (m, 30H), 3.49 (d, J = 5.8 Hz, 4H), 2.41 (t, J = 2.4 Hz, 1H), 2.13 (sept, J = 5.8 Hz, 1H), 1.08–1.03 (m, 42H) ppm. ESI-TOF MS: m/z: calculated for $C_{41}H_{84}NaO_{11}Si_2$: 831.5450 [M + Na]$^+$; found: 831.5517.

Synthesis of 8. To a dry DMF (70 mL) and *N*,*N*-diisopropylethylamine (0.070 mL, 0.42 mmol) suspension of **7** (0.150 g, 0.229 mmol), CuI (10.3 mg, 0.054 mmol) and $Pd(PPh_3)_4$ (26.6 mg, 0.023 mmol) was added a dry DMF (8 mL) solution of **6** (0.339 g, 0.456 mmol) dropwise over 10 min at 25 °C under Ar, and the resulting mixture was stirred overnight at 50 °C. The reaction mixture was further stirred for 4 h at 70 °C, which was then cooled to 25 °C followed by evaporation under reduced pressure at 50 °C. To the residue was added brine (40 mL) and the mixture was extracted with with EtOAc (50 mL, three times). The collected organic extract was dried over anhydrous Na_2SO_4, and filtered off from insoluble substances. The filtrate was evaporated to dryness under reduced pressure at 40 °C, and the residue was chromatographed on silica gel (Silica Gel 60) with a gradient of AcOEt/*n*-hexane (50/50 to 100/0 v/v) to allow isolation of **8** as yellowish oil in 93% yield. ^{1}H NMR (400 MHz, $CDCl_3$ containing 0.03% TMS, 22 °C): δ 8.2 (broad s, 1H), 7.94 (s, 1H), 7.68 (m, 2H), 7.7–7.2 (m, 7H), 6.84 (d, J = 8.8 Hz, 4H), 6.25 (dd, J = 7.6 and 6.0 Hz, 1H), 4.49 (m, 1H), 4.11 (d, J = 4.0 Hz, 2H), 4.05 (quart, J = 3.2 Hz, 2H), 3.83 (t, J = 5.6 Hz, 4H), 3.79 (s, 6H), 3.7–3.5 (m, 28H), 3.45 (m, 6H), 3.39 (m, 2H), 2.47 (m, 1H), 2.27 (m, 1H), 2.13 (m, 1H), 1.10–1.05 (m, 42H) ppm. ESI-TOF MS: m/z: calculated for $C_{71}H_{112}N_2NaO_{18}Si_2$: 1359.7346 [M + Na]$^+$; found: 1359.7316.

Synthesis of 9. To a dry CH_2Cl_2 (25 mL) solution of **8** (evaporated twice from dry CH_2Cl_2 solution just prior to use; 0.306 g, 0.230 mmol) were added *N*,*N*-diisopropylethylamine (0.16 mL, 0.92 mmol) and 2-cyanoethyl *N*,*N*-diisopropylchlorophosphoramidite (0.060 mL, 0.27 mmol) at 0 °C under Ar, and the reaction mixture was stirred for 40 min at 0 °C and then for 4 h at 25 °C. To the reaction mixture was added MeOH (0.5 mL), and the resulting mixture was evaporated to dryness under reduced pressure at 25 °C. The residue was chromatographed on silica gel (Chromatorex NH silica) with a gradient of CH_2Cl_2/MeOH (98/2 to 96/4 v/v) to allow isolation of **9** (0.173 g, 0.113 mmol) as pale yellow oil in 49%

yield. ^{1}H NMR (400 MHz, $CDCl_3$ containing 0.03% TMS, 25 °C): δ 8.05 and 8.01 (s and s, total 1H), 7.67 (d and d, J = 8.4 and 8.4 Hz, total 2H), 7.58–7.25 (m, 7H), 6.83 (dd, J = 8.8 and 4.0 Hz, 4H), 6.25 (dd, J = 7.6 and 6.0 Hz, 1H), 4.60 (m, 1H), 4.17 (m, 2H), 3.97 (m, 2H), 3.9–3.4 (m, 46H), 3.27 (m, 1H), 2.63 (m, 4H), 2.57 (m, 1H), 2.45 (m, 1H), 2.28 (m, 1H), 2.12 (m, 1H), 1.18–1.14 (m, 12H), 1.10–1.05 (m, 42H) ppm. ESI-TOF MS: m/z: calculated for $C_{80}H_{129}N_4NaO_{19}PSi_2$: 1559.8425 $[M + Na]^+$; found: 1559.8416.

Synthesis of Tribranched TEG modified deoxythymine

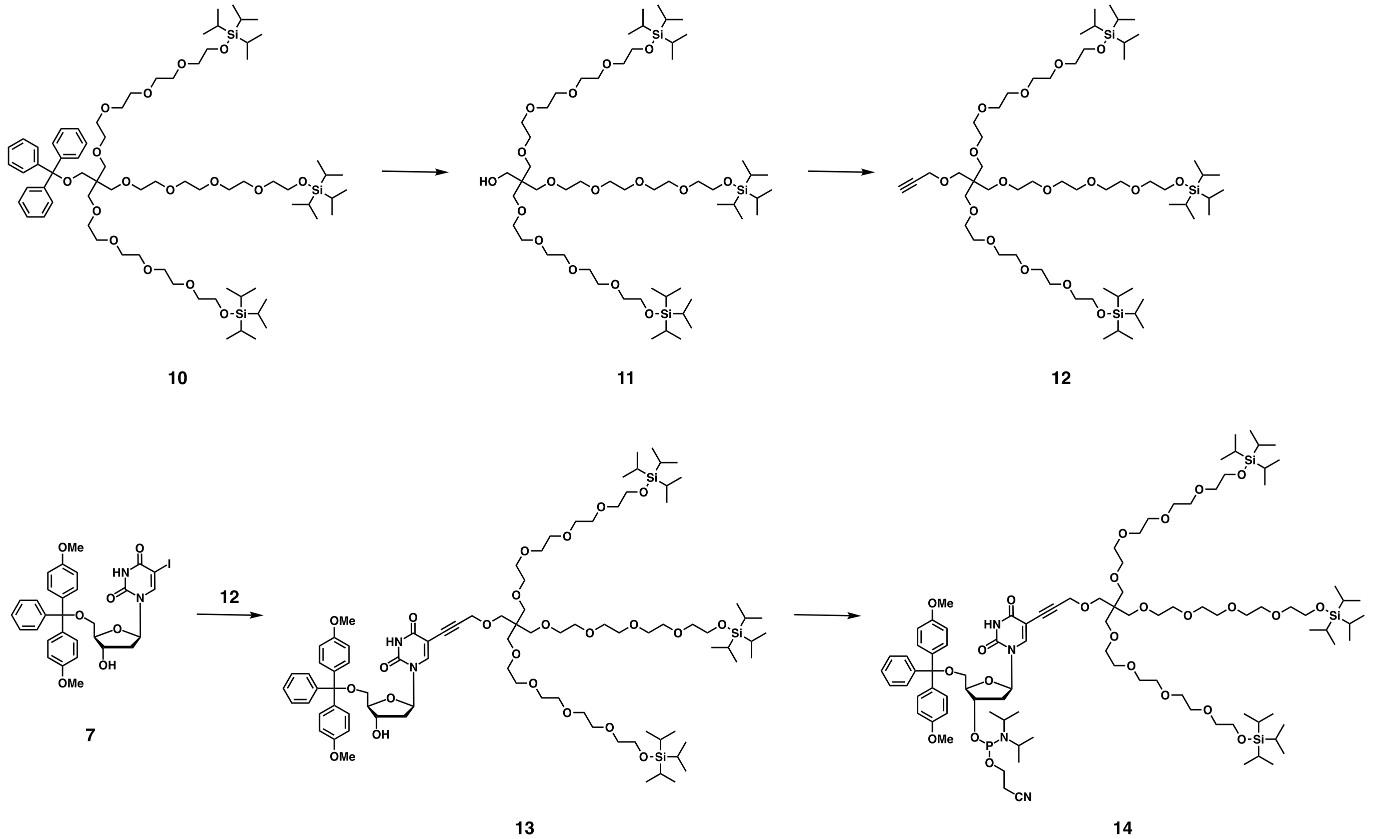

Tribranched TEG modified deoxythymine **14** was synthesized by following the synthetic procedure of Dibranched TEG modified deoxythymine **9** .

10: ^{1}H NMR (400 MHz, $CDCl_3$ containing 0.03% TMS, 23 °C): δ 7.42 (m, 6H), 7.29 (m, 6H), 7.23 (m, 3H), 3.83 (t, J = 5.6 Hz, 6H), 3.7–3.5 (m, 48H), 3.15 (d, J = 5.6 Hz, 2H), 1.10–1.05 (m, 63H) ppm. ESI-TOF MS: m/z: calculated for $C_{75}H_{134}NaO_{16}Si_3$: 1397.8877 $[M + Na]^+$; found: 1397.8851.

11: ^{1}H NMR (400 MHz, $CDCl_3$ containing 0.03% TMS, 29 °C): δ 3.83 (t, J = 5.8 Hz, 6H), 3.74 (dd, J = 4.8 and 5.8 Hz, 2H), 3.7–3.5 (m, 48H), 2.87 (t, J = 4.8 Hz, 1H), 1.08–1.03 (m, 63H) ppm. ESI-TOF MS: m/z: calculated for $C_{56}H_{120}NaO_{16}Si_3$: 1155.7782 $[M + Na]^+$; found: 1155.7772.

12: ^{1}H NMR (400 MHz, $CDCl_3$ containing 0.03% TMS, 24 °C): δ 4.11 (d, J = 2.4 Hz, 2H), 3.83 (t, J = 5.6 Hz, 6H), 3.7–3.5 (m, 44H), 3.49 (d, J = 5.8 Hz, 6H), 2.41 (t, J = 2.4 Hz, 1H), 1.08–1.03 (m, 63H) ppm. ESI-TOF MS: m/z: calculated for $C_{59}H_{122}NaO_{16}Si_3$: 1193.7938 $[M + Na]^+$; found: 1193.7904.

13: ^{1}H NMR (400 MHz, $CDCl_3$ containing 0.03% TMS, 22 °C): δ 8.2 (broad s, 1H), 7.94 (s, 1H), 7.68 (m, 2H), 7.7–7.2 (m, 7H), 6.84 (d, J = 8.8 Hz, 4H), 6.25 (dd, J = 7.6 and 6.0 Hz, 1H), 4.49 (m, 1H), 4.11 (d, J = 4.0 Hz, 2H), 4.05 (quart, J = 3.2 Hz, 2H), 3.83 (t, J = 5.6 Hz, 6H), 3.79 (s, 6H), 3.7–3.5 (m, 42H), 3.45 (m, 8H), 3.39 (m, 2H), 2.47 (m, 1H), 2.27 (m, 1H), 1.10–1.05 (m, 63H) ppm. ESI-TOF MS: m/z: calculated for $C_{89}H_{150}N_2NaO_{23}Si_2$: 1721.9835 $[M + Na]^+$; found: 1721.9848.

14: ^{1}H NMR (400 MHz, $CDCl_3$ containing 0.03% TMS, 25 °C): δ 8.05 and 8.01 (s and s, total 1H), 7.67 (d and d, J = 8.4 and 8.4 Hz, total 2H), 7.58–7.25 (m, 7H), 6.83 (dd, J = 8.8 and 4.0 Hz, 4H), 6.25 (dd, J = 7.6 and 6.0 Hz, 1H), 4.60 (m, 1H), 4.17 (m, 2H), 3.97 (m, 2H), 3.9–3.4 (m, 62H), 3.27 (m, 1H), 2.63 (m, 6H), 2.57 (m, 1H), 2.45 (m, 1H), 2.28 (m, 1H), 1.18–1.14 (m, 12H), 1.10–1.05 (m, 63H) ppm. ESI-TOF MS: m/z: calculated for $C_{98}H_{167}N_4NaO_{24}PSi_3$: 1922.0913 $[M + Na]^+$; found: 1922.0917.

The synthesis of oligonucleotides containing TEG-modified deoxythymines

The oligonucleotides with TEG-modified deoxythymines were synthesized using standard phosphoramidite methods on an automated DNA synthesizer. The amidites of TEG-modified deoxythymines were incorporated during synthesis, and the obtained oligonucleotides were purified by Japan Bio Services Co., LTD, Japan. To remove the protecting groups, the oligonucleotides were incubated in 30% (v/v) ammonia solution for 8 h at 55 ºC. The triisopropylsilyl (TIPS) group was removed in tetra-*n*-butylammonium fluoride (TBAF) solution. The oligonucleotides were desalted with Quick-Precip Plus Solution (Edge Bio Co.) and purified by HPLC. The molecular weights of the oligonucleotides were confirmed by MALDI-TOF MS.

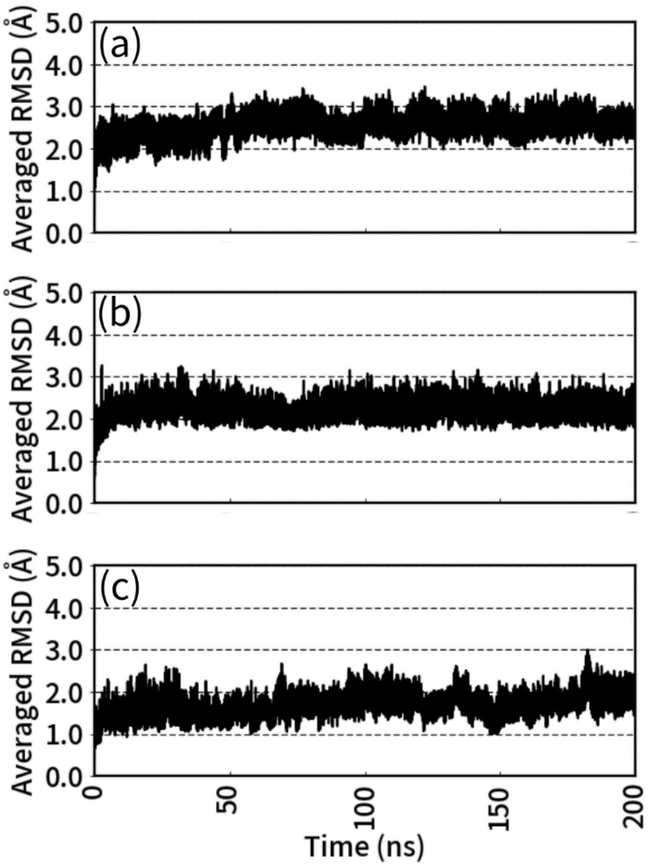

Figure S1. Change in root mean square deviation (RMSD) values for heavy atoms of backbone in (a) Q1, (b) Q1-$(X4)_4$ and (c) Q1-$(2X4)_4$.

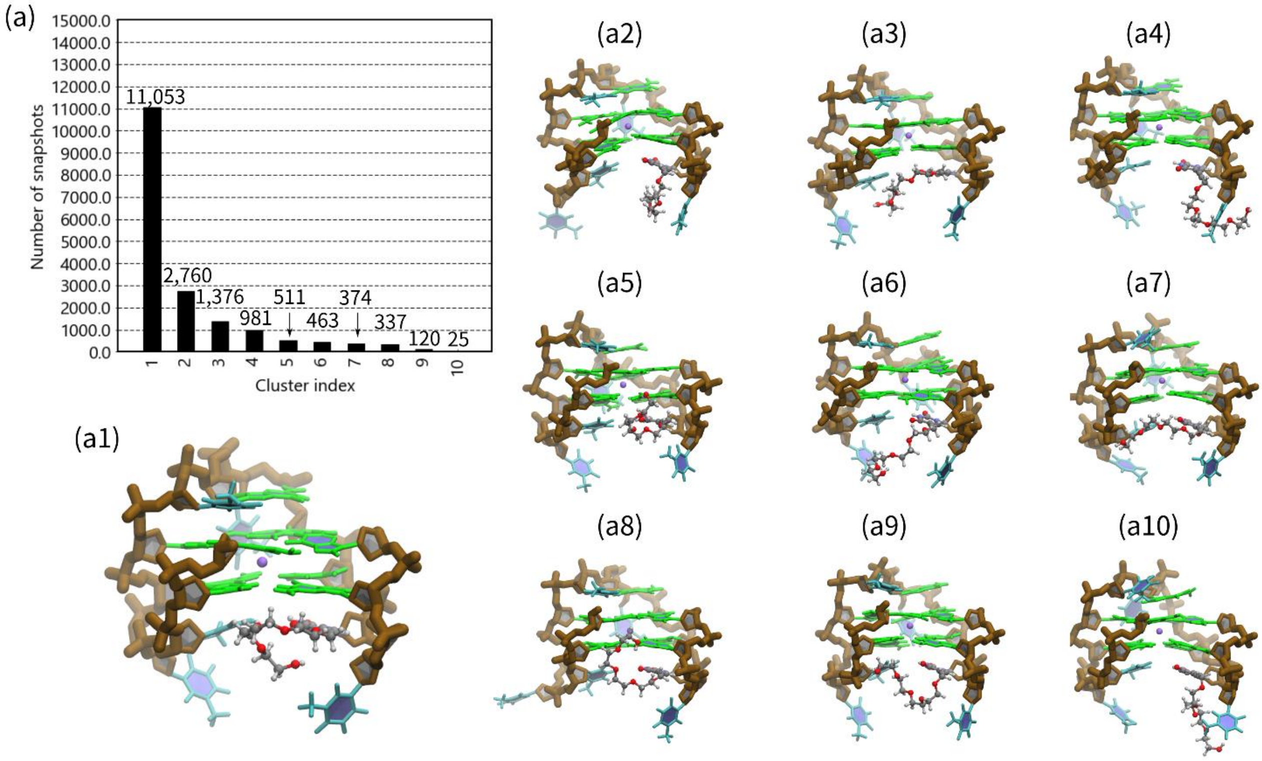

Figure S2. Hierarchical clustering of snapshots during simulations of Q1-$(X4)_4$. (a) The number of snapshots in each cluster. (b) Representative conformation for each cluster (cluster 1 is shown in Figure 3b) of Q1-$(X4)_4$. The number on each conformation specifies cluster number. Backbone is shown in dark green, and guanine and thymine bases are shown in green and cyan blocks, respectively. Potassium ions are represented by violet balls. All atoms of (X4) are shown in ball and stick representation with red for oxygen, blue for nitrogen, gray for carbon, and white for hydrogen.

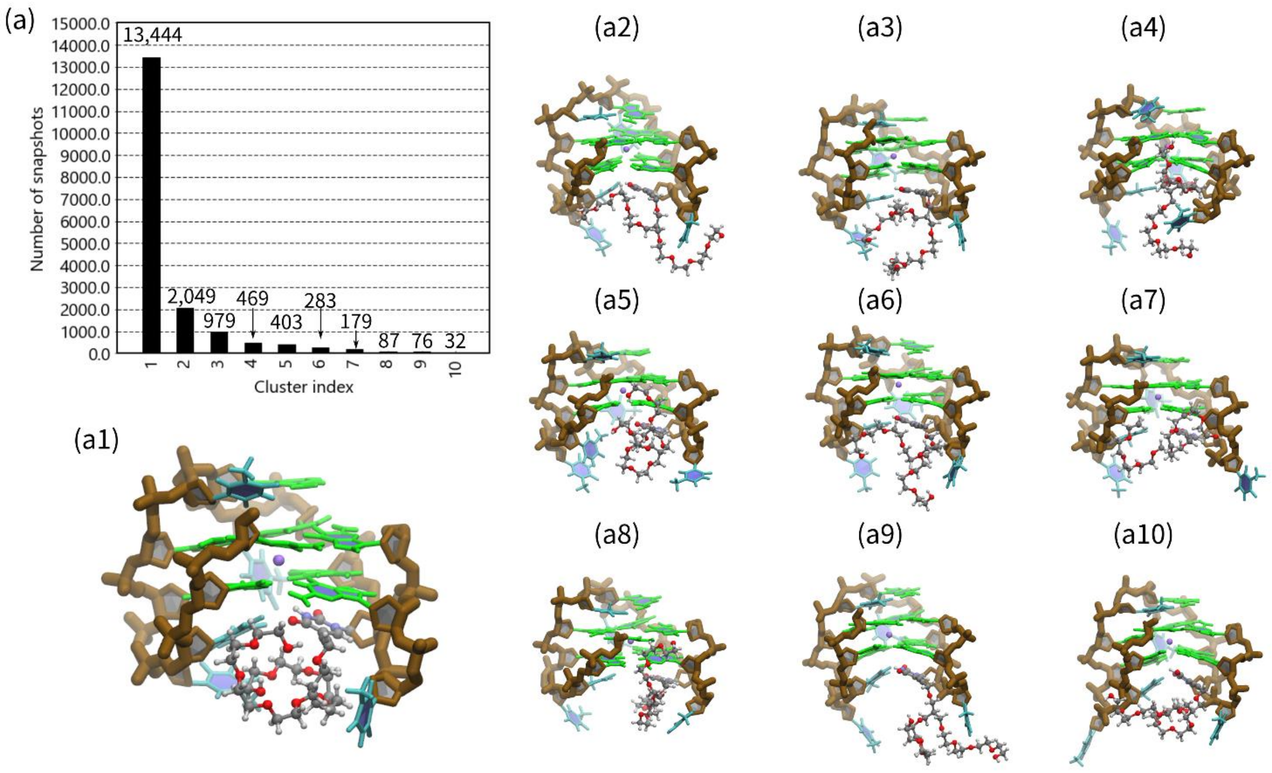

Figure S3. Hierarchical clustering of snapshots during simulations of Q1-$(2X4)_4$. (a) The number of snapshots in each cluster. (b) Representative conformation for each cluster (cluster 1 is shown in Figure 3c) of Q1-$(2X4)_4$. The number on each conformation specifies cluster number. Backbone is shown in dark green, and guanine and thymine bases are shown in green and cyan blocks, respectively. Potassium ions are represented by violet balls. All atoms of (2X4) are shown in ball and stick representation with red for oxygen, blue for nitrogen, gray for carbon, and white for hydrogen.

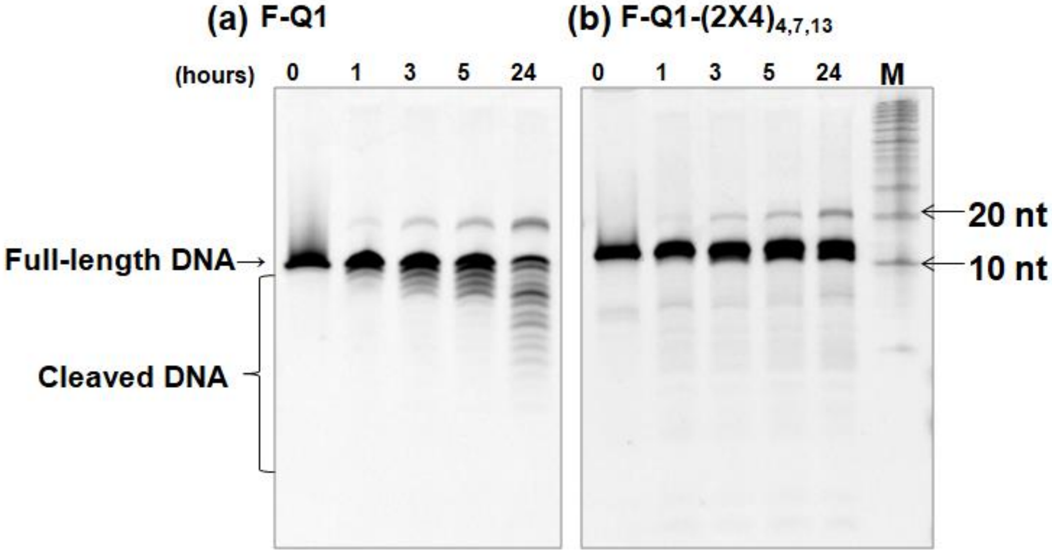

Figure S4. Denaturing gel electrophoresis of (a) F-Q1 and (b) F-Q1-$(2X4)_{4,,7,13}$ after the addition of human serum at 37 °C. Samples incubated for 0, 1, 3, 5, and 24 hours were loaded on lanes 1 to 5, respectively. Lane M shows 10-nt size marker. After electrophoresis, the gels were stained by SYBR® Gold (PerkinElmer Life Sciences).

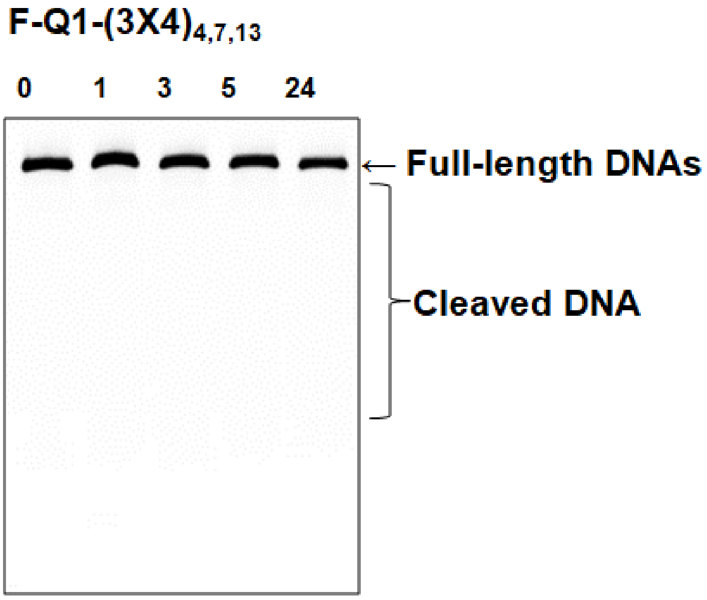

Figure S5. Denaturing gel electrophoresis of F-Q1-$(3X4)_{4,,7,13}$ after the addition of human serum at 37 °C. Samples incubated for 0, 1, 3, 5, and 24 hours were loaded on lanes 1 to 5, respectively.
